# Cachinnate Vehemently

**Published:** 1854-05

**Authors:** 


### CACHINNATE VEHEMENTLY.

The Editor has found the above to be a necessary prescription in some instances; the material therefor having to be looked up by the patient: it may sometimes occur, however, that the ingredients necessary to produce the effect, are not in any market within the patient's reach. Sometimes, however, in fact not unfrequently, a remedy, which acts admirably on number one, has no effect at all on number two, while on number three it aggravates all the symptoms which were intended to be removed, so that the article here given, is not offered as infallible. In case, however, of over action, the best antidote is extremely simple, for the patient has only to try and shut his mouth, or take a teaspoonful of green persimmon.

